# Clinical Progress and Preclinical Insights Into Umbilical Cord Blood Transplantation Improvement

**DOI:** 10.1093/stcltm/szac056

**Published:** 2022-08-16

**Authors:** Zhongjie Sun, Bing Yao, Huangfan Xie, XunCheng Su

**Affiliations:** State Key Laboratory of Elemento-organic chemistry, College of Chemistry, Nankai University, Tianjin, People’s Republic of China; Newish Technology (Beijing) Co., Ltd., Beijing, People’s Republic of China; Zhejiang Hisoar Pharmaceutical Co., Ltd., Taizhou, Zhejiang Province, People’s Republic of China; School of Pharmaceutical Sciences, Key Laboratory of Bioorganic Phosphorus Chemistry and Chemical Biology (Ministry of Education), Tsinghua University, Beijing, People’s Republic of China; Newish Technology (Beijing) Co., Ltd., Beijing, People’s Republic of China; State Key Laboratory of Elemento-organic chemistry, College of Chemistry, Nankai University, Tianjin, People’s Republic of China

**Keywords:** umbilical cord blood, hematopoietic stem and progenitor cells, engraftment, ex vivo expansion, homing, clinical trial

## Abstract

The application of umbilical cord blood (UCB) as an important source of hematopoietic stem and progenitor cells (HSPCs) for hematopoietic reconstitution in the clinical context has steadily grown worldwide in the past 30 years. UCB has advantages that include rapid availability of donors, less strict HLA-matching demands, and low rates of graft-versus-host disease (GVHD) versus bone marrow (BM) and mobilized peripheral blood (PB). However, the limited number of HSPCs within a single UCB unit often leads to delayed hematopoietic engraftment, increased risk of transplant-related infection and mortality, and proneness to graft failure, thus hindering wide clinical application. Many strategies have been developed to improve UCB engraftment, most of which are based on 2 approaches: increasing the HSPC number ex vivo before transplantation and enhancing HSPC homing to the recipient BM niche after transplantation. Recently, several methods have shown promising progress in UCB engraftment improvement. Here, we review the current situations of UCB manipulation in preclinical and clinical settings and discuss challenges and future directions.

## Graphical Abstract

**Figure:**
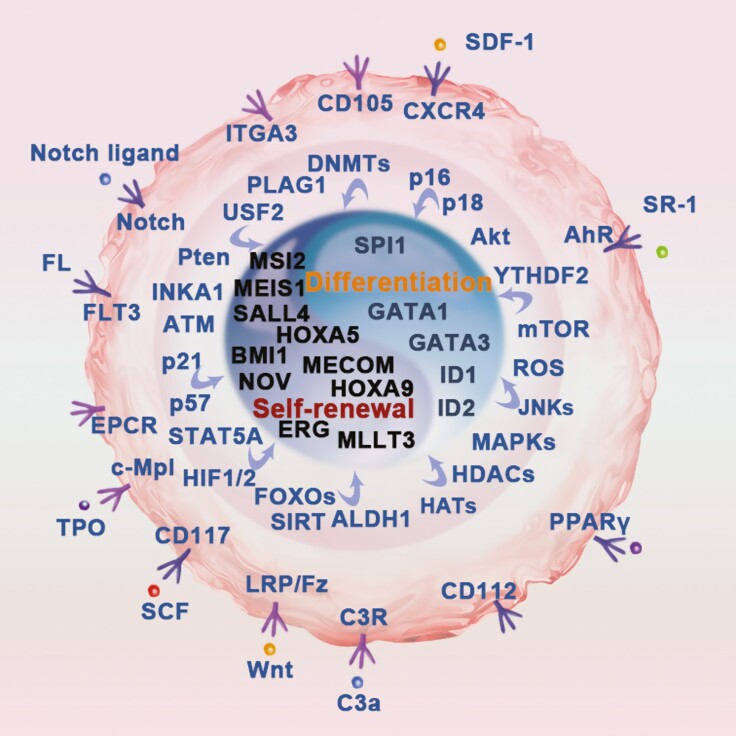

**Significance Statement**

Many strategies have been developed to improve UCB engraftment, most of which are based on 2 approaches: increasing the HSPC number ex vivo before transplantation and enhancing HSPC homing to the recipient BM niche after transplantation. Recently, several methods have shown promising progress in UCB engraftment improvement. This article summarizes clinical trials on UCB transplantation improvement from the earliest to the latest publications, covering ex vivo expansion and promotion of homing. The latest preclinical studies on UCB engraftment improvement by various approaches are also discussed, which are not available in other recently published reviews.

## Introduction

Hematopoietic stem cells (HSCs) are a type of adult stem cell that can provide a steady lifelong pool of various hematopoietic and immune cells.^1^ HSC transplantation (HSCT) has been applied clinically to treat more than 80 diseases, including hematological malignancies and disorders.^2,3^ There are approximately 70 000 cases of HSCT worldwide annually.^4^ The most commonly used HSC sources are mobilized peripheral blood (PB) and to a lesser extent bone marrow (BM).^5^ Nevertheless, wide application of PB- and BM-based HSCT has been hindered by the strict demand for HLA matching and severe GVHD.^6^ Umbilical cord blood (UCB), with less stringent HLA-matching demand, ready availability, and lower risk of graft-versus-host disease (GVHD), has become an alternative source for HSCT.^7^

UCB HSCT has been developed for 30 years, and it is estimated that the total number of UCB HSCT cases worldwide exceeds 45 000,^7^ and the number of cases of UCB HSCT is still growing. Overall, approximately 800 000 and 4 000 000 UCB units have been stored in public and private banks, respectively.^8^ However, limitations of UCB HSCT are that a relatively low number of HSPCs in a single UCB unit cannot meet the demand for adult HSCT and that hematopoietic and immune recovery after UCB HSCT is delayed, resulting in a high risk of infection and mortality; in addition, UCB HSCT is more prone to graft failure than BM or mobilized PB transplantation.^9^ Although double UCB units and improved healthcare might alleviate such problems, the cost can be prohibitive. Indeed, the acquisition cost of a single UCB is $30 000-60 000, not including inpatient costs.^10,11^

**Figure 1.**
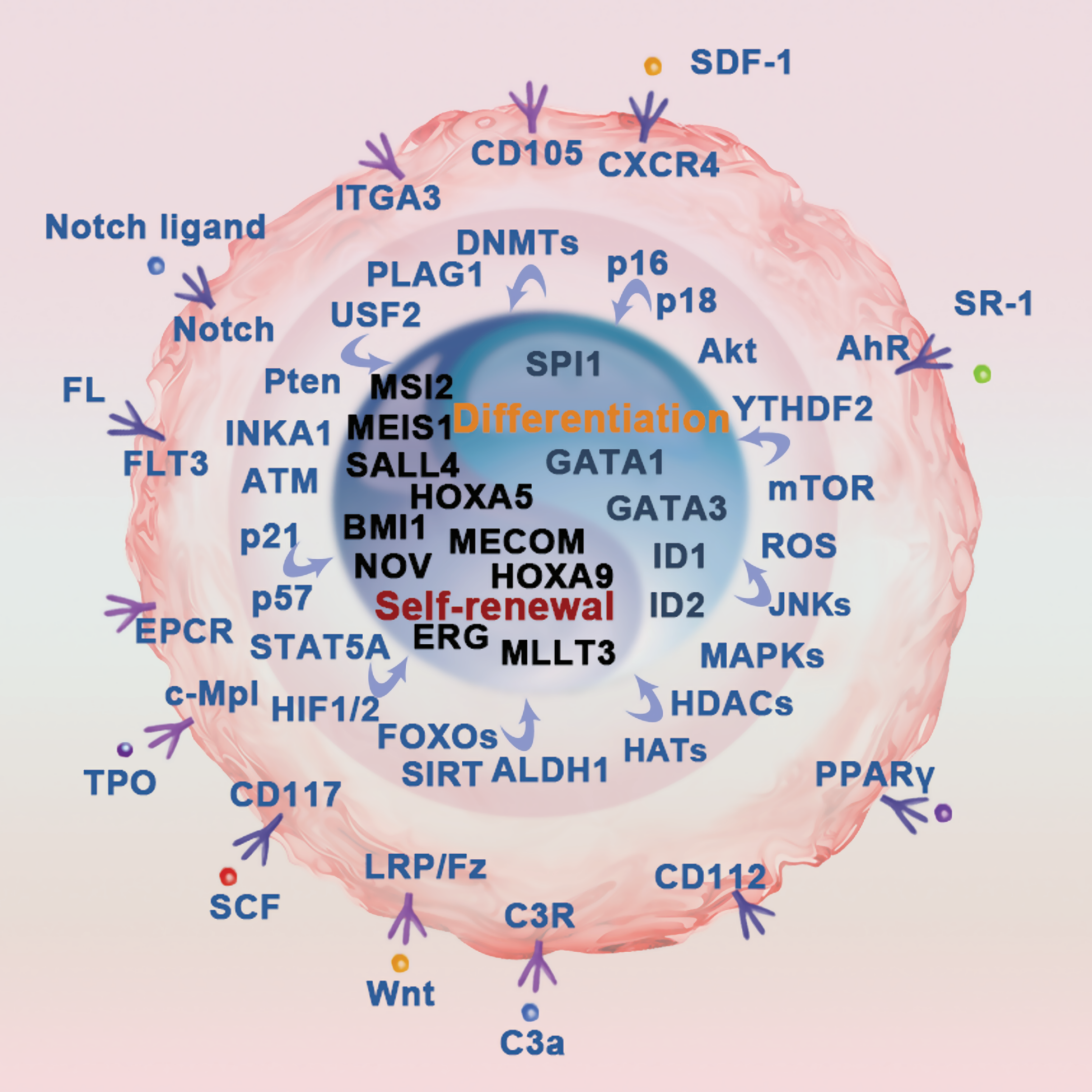
Extrinsic and intrinsic factors that regulate HSC self-renewal and differentiation. Various extrinsic and intrinsic factors have been identified to regulate human HSC self-renewal and differentiation; however, the regulatory core for HSC self-renewal and differentiation remains incompletely understood. This picture highlights selected but not all regulators of human HSC self-renewal and differentiation. In brief, extracellular stimuli act on the surface receptor; the signals are passaged by a series of effectors to the core regulator, where extrinsic and intrinsic signals converge. The cell fate is then determined depending on the strength of the supposed self-renewal regulatory core and differentiation regulatory core (as shown in the Tai Chi diagram in the figure).

To date, there have been many efforts to improve UCB engraftment, with mainly 2 approaches: expanding the UCB HSPC number via ex vivo expansion and improving HSC homing to the recipient’s BM after transplantation. Both strategies are based on the insight of HSC self-renewal regulators and niche factors identified to affect HSC behaviors, which have been discussed in detail in previous reviews.^12-14^ These factors exert their functions by converging cues at the regulatory core of HSC self-renewal and differentiation (Fig. 1).^15,16^ This review focuses on the latest clinical trials of UCB transplantation and discusses the progress of preclinical studies, hoping to inspire future improvements in UCB engraftment.

### Clinical Trials on UCB Engraftment Improvement

Clinical trials on UCB engraftment improvement are usually based on preclinical studies showing benefits for engraftment after certain manipulations. As HSC markers have yet to be defined, these studies have regarded CD34^+^ cells or CD133^+^ cells, a heterogeneous population containing long-term repopulating HSCs, as putative HSCs.^17-20^ Therefore, functional assessment of these cells after manipulation is of great importance. In preclinical studies, limiting dilution assay (LDA) in transplantation models is used to count the number of HSCs among input cells, whereby long-term HSCs are able to reconstitute in irradiated immunodeficient recipient NOD/SCID mice (or subsequent adaptations of this mouse strain, for instance, NSG and NOG mice) for more than 24 weeks, with both lymphoid and myeloid repopulation.^21^ In general, UCB engraftment kinetics in patient recipients are slow in the absence of manipulation. The median time to neutrophil and platelet recovery after double UCB transplantation (UCBT) is approximately 27 and 48 days, respectively, twice that of BM and mobilized PB HSCT.^22,23^ Therefore, the most important aim of UCB manipulation is to shorten the median time to engraftment. Tables 1 and 2 summarize clinical trials on UCBT improvement.

**Table 1.** List of clinical trials on UCB manipulation.

Manipulation	Strategy	Conditions	ClinicalTrials.gov identifier	Status	Locations
Expansion					
Cytokines	Single UCB: one portion with CD34^+^ cells selected and expanded; the other portion unmanipulated	Hematologic malignancies	NA	NA; Completed	University of Colorado, Denver, Colorado, US
UM171	Single UCB with CD34^+^ cells selected and expanded; CD34^-^ cells are also infused	Hematologic malignancies	NCT02668315	Phase I/II; Completed	Hopital Maisonneuve-Rosemont, Montreal, Quebec, Canada
UM171	Single UCB with CD34^+^ cells selected and expanded; CD34^−^ cells are also infused	High-risk hematological malignancies	NCT04103879	Phase II; Recruiting	Fred Hutchinson/University of Washington Cancer Consortium, Seattle, Washington, US
UM171	Single UCB with CD34^+^ cells selected and expanded; CD34^−^ cells are also infused	Multiple myeloma	NCT03441958	Phase I/II; Recruiting	CIUSSS de l’Est-del’île-de-Montréal, Installation Hôpital Maisonneuve Rosemond, Montréal, Quebec, Canada
UM171	Single UCB with CD34^+^ cells selected and expanded; CD34^−^ cells are also infused	High-risk hematological malignancies	NCT03913026	Phase II; Recruiting	CIUSSS de l’Est-del’île-de Montreal, Hôpital Maisonneuve-Rosemont, Montréal, Quebec, Canada
UM171	Single UCB with CD34^+^ cells selected and expanded; CD34^−^ cells are also infused	High-risk myeloid malignancies	NCT04990323	Phase I/II; Recruiting	Memorial Sloan Kettering Cancer Center, New York, New York, US
UM171	Single UCB with CD34^+^ cells selected and expanded; CD34^−^ cells are also infused	Sickle cell disease	NCT04594031	Phase I; Recruiting	Stanford University School of Medicine, Palo Alto, California, US; University of California San Francisco, San Francisco, California, US
SR-1	Double UCB; one with CD34^+^ cells selected and expanded; CD34^−^ cells are also infused; the other unmanipulated	Hematologic malignancies	NCT01474681	Phase I/II; Completed	Novartis Investigative Site, Minneapolis, Minnesota, US
SR-1	Double UCB; one with CD34^+^ cells selected and expanded; CD34^−^ cells are also infused; the other unmanipulated	Inherited metabolic disorders	NCT03406962	Phase II; Completed	University of Minnesota, Minneapolis, Minnesota, US; Cincinnati Children’s Hospital Medical Center, Cincinnati, Ohio, US
Nicotinamide	Double UCB; one with AC133^+^ cells selected and expanded; AC133^−^ cells are also infused; the other unmanipulated	Hematological malignancies	NCT01221857	Phase I/II; Completed	Loyola University, Cardinal Bernardin Cancer Center, Maywood, Illinois, US; Duke University Medical Center, Durham, North Carolina, US
Nicotinamide	Single UCB with AC133^+^ cells selected and expanded; AC133^−^ cells are also infused	Hematological malignancies	NCT01816230	Phase I/II; Completed	Multicenter in US, Italy, Netherlands, Singapore and Spain
TEPA	Single UCB with expanded and unmanipulated portions	Hematologic malignancies	NCT00469729	Phase II/III; Completed	Multicenter in US, Hungary, Israel, Italy and Spain
TEPA	Single UCB with expanded and unmanipulated portions	Hematologic malignancies	NCT01484470	Phase II; Completed	Loyola University Medical Center, Maywood, Illinois, US
Valproic Acid	Double UCB; one with CD34^+^ cells selected and expanded; CD34^−^ cells are also infused; the other is unmanipulated	Hematologic malignancies	NCT03885947	Phase I; Completed	Icahn School of Medicine at Mount Sinai, New York, New York, US
Delta1ext-IgG	Double UCB; one with CD34^+^ cells selected and expanded; the other is unmanipulated	Hematologic malignancies	NA	Phase I; Completed	Fred Hutchinson Cancer Research Center, Seattle, Washington, US
MSC	Double UCB; one expanded on MSC, the other is unmanipulated	Myelodysplastic syndrome; Leukemia	NCT00498316	Phase I; Completed	University of Texas MD Anderson Cancer Center, Houston, Texas, US
MSC	Double UCB; one expanded on MSC, the other is unmanipulated	Hematological malignancies	NCT01624701	Phase I/II; Terminated (Funding expired)	Singapore General Hospital, Singapore
Homing					
C3a	Double UCB; one primed with C3a, the other is unmanipulated	Hematological malignancies	NCT00963872	Phase I/II; Terminated (Lack of efficacy after interim analysis)	University of Minnesota Medical Center—Fairview, Minneapolis, Minnesota, US
Sitagliptin	Single UCB with sitagliptin taken orally	Hematological malignancies	NCT00862719	Phase II; Completed	IU Simon Cancer Center, Indianapolis, Indiana, US
Sitagliptin	Single UCB with sitagliptin taken orally	Hematological malignancies	NCT01720264	Phase II; Completed	Indiana University Melvin and Bren Simon Cancer Center, Indianapolis, Indiana, US; New York Medical College/Westchester Medical Center/Maria Fareri Children’s Hosptial, Valhalla, New York, US
PGE2	Single FT1050-treated UCB unit	Hematological malignancies	NCT01527838	Phase I; Completed	Massachusetts General Hospital, Boston, Massachusetts, US; Dana Farber Cancer Institute-Hematopoietic Stem Cell Transplant Program, Boston, Massachusetts, US; Ohio State University Comprehensive Cancer Center, Columbus, Ohio, US
PGE2	Single FT1050-treated UCB unit	Metabolic disorders	NCT02354443	Phase I; Terminated (Business decision)	Boston Children’s Hospital, Boston, Massachusetts, US; Duke University Medical Center, Durham, North Carolina, US
PGE2	Single FT1050-treated UCB unit	Hematologic malignancies	NCT02354417	Phase I; Terminated	City of Hope, Duarte, California, US; Boston Children’s Hospital, Boston, Massachusetts, US
PGE2	Double UCB; one with FT1050 treatment; the other is unmanipulated	Hematologic malignancies	NCT00890500	Phase I; Completed	Massachusetts General Hospital, Boston, Massachusetts, US; Dana-Farber Cancer Institute, Boston, Massachusetts, US
PGE2	Double UCB; one with FT1050 treatment; the other is unmanipulated	Hematologic malignancies	NCT01627314	Phase II; Terminated (Business decision)	Multicenter in US
Fucosylation	Double UCB; one is fucosylated; the other is unmanipulated	Hematologic malignancies	NCT01471067	Phase I; Completed	University of Texas MD Anderson Cancer Center, Houston, Texas, US
Hyperbaric Oxygen	Double UCB with administration of hyperbaric oxygen	Hematologic malignancies	NCT02099266	NA; Completed	University of Kansas Medical Center, Kansas City, Kansas, US
IBMT	Intrabone infusion of double UCB	Hematological malignancies	NCT00886522	Phase II; Completed	Hematology Institute “L. and A. Seràgnoli”, S. Orsola-Malpighi University Hospital, Bologna, Italy
Combination					
MSC and fucosylation	Double UCB; one expanded on MSC and then fucosylated; the other is unmanipulated	Hematologic malignancies	NCT03096782	Phase II; Recruiting	MD Anderson Cancer Center, Houston, Texas, US

Abbreviations: C3a, completement 3a fragment; IBMT, intrabone marrow transplant; MSC, mesenchymal stromal cell; NA, not applicable; SR-1, stem-regenin 1; PGE2, prostaglandin E2; TEPA, tetraethylenepentamine; UCB, umbilical cord blood.

**Table 2.** Summary of clinical trial data of UCB manipulation.

Treatment	Agonist	Culture period	Expansion fold	Cell dose (cells/kg recipient weight)			Hematopoietic engraftment (number of patients)	Day of engraftment	Possible mechanism
				Unmanipulated	Manipulated	Total			
Cytokines^24^	SCF, G-CSF, MGDF	10 d	TNC: 56.0 CD34^+^: 4.0	TNC: 1.2 × 10^7^; CD34^+^: 7.4 × 10^4^	NA	TNC: 1.0 × 10^7^; CD34^+^: 10.4 × 10^4^	30/30	Neutrophils: 28 d. Platelets: 106 d	Promotion of self-renewal and prevention of differentiation
Copper chelator^25^	TEPA, SCF, FL, IL-6, TPO	21 d	TNC: 219.0 CD34^+^: 6.0	TNC: 1.7 × 10^7^; CD34^+^: 5.5 × 10^4^	TNC: 4.4 × 10^6^; CD34^+^: 65.1 × 10^4^	NA	9/10	Neutrophils: 30 d. Platelets: 48 d	Unclear
Copper chelator^26^	TEPA, SCF, FL, IL-6, TPO	21 d	TNC: 400.0 CD34^+^: 77.0	TNC: 2.4 × 10^7^; CD34^+^: 23.8 × 10^5^	TNC: 0.9 × 10^7^; CD34^+^: 94.2 × 10^5^	NA	85/101	Neutrophils: 21 d. Platelets: 54 d	Unclear
IBMT^27^	NA	NA	NA	TNC: 2.6 × 10^7^; CD34^+^: 10.0 × 10^4^	NA	TNC: 2.6 × 10^7^; CD34^+^: 10.0 × 10^4^	28/28	Neutrophils: 23 d. Platelets: 36 d	Increased homing to bone marrow
Notch ligand^28^	Delta1^ext-IgG^, Fibronectin, SCF, TPO, FL, IL-3, IL-6	16 d	TNC:562.0 CD34^+^: 164.0	TNC: 3.3 × 10^7^; CD34^+^: 24.0 × 10^4^	TNC: 4.6 × 10^7^; CD34^+^: 603.0 × 10^4^	NA	7/10	Neutrophils: 16 d. Platelets: NA	Activation of Notch signaling, which induces self-renewal
MSC coculture^29^	SCF, TPO, FL, G-CSF	14 d	TNC:12.2 CD34^+^: 30.1	TNC: 2.3 × 10^7^; CD34^+^: 4.0 × 10^4^	TNC: 5.8 × 10^7^; CD34^+^: 1.0 × 10^6^	TNC: 8.3 × 10^7^; CD34^+^: 1.2 × 10^6^	23/24	Neutrophils: 15 d. Platelets: 42 d	Secretion of SDF-1, which prevents differentiation
dmPGE2 priming^30^	dmPGE2	2 h	NA	TNC: 1.7 × 10^7^; CD34^+^: 6.0 × 10^4^	TNC: 1.8 × 10^7^; CD34^+^: 7.0 × 10^4^	NA	10/12	Neutrophils: 17.5 d. Platelets: 43 d	Enhanced Wnt signaling and homing
C3a priming^31^	C3a	15 minutes	NA	TNC: 2.5 × 10^7^; CD34^+^: 3.4 × 10^5^	TNC: 1.5 × 10^7^; CD34^+^: 3.2 × 10^5^	TNC: 4.0 × 10^7^; CD34^+^: 7.6 × 10^5^	21/29	Neutrophils: 7 d. Platelets: NA	Activation of CXCR4, which facilities homing
DPP4 inhibition^32^	Sitagliptin	NA	NA	NA	TNC: 2.4 × 10^7^; CD34^+^: 1.0 × 10^5^	NA	17/17	Neutrophils: 21 d. Platelets: NA	Strengthens the SDF-1-CXCR4 axis, which facilities homing
Nicotinamide^33^	Nicotinamide, SCF, TPO, IL-6, FBS	21 d	TNC:486.0 CD34^+^: 72.0	TNC: 2.6 × 10^7^; CD34^+^: 0.6 × 10^5^	TNC: 2.5 × 10^7^; CD34^+^: 3.5 × 10^6^	NA	10/11	Neutrophils: 13 d. Platelets: 33 d	Decreased differentiation by inhibiting SIRT1 deacetylase
Nicotinamide^34^	Nicotinamide, SCF, TPO, IL-6, FBS	21 d	TNC:NA CD34^+^: 33.0	NA	TNC: 4.9 × 10^7^; CD34^+^: 6.3 × 10^6^	NA	34/36	Neutrophils: 11.5 d. Platelets: 34 d	Decreased differentiation by inhibiting SIRT1 deacetylase
Fucosylation^35^	Fucosyltransferase-VI, guanosine diphosphate fucose	30 minutes	NA	TNC: 2.6 × 10^7^; CD34^+^: 1.2 × 10^5^	TNC: 1.8 × 10^7^; CD34^+^: 0.9 × 10^5^	TNC: 4.26 × 10^7^; CD34^+^: 2.4 × 10^5^	20/22	Neutrophils: 17 d. Platelets: 35 d	Increased binding of E- and P-selectins, which facilitates homing
SR-1^36^	SR-1, SCF, TPO, FL, IL-6	15 d	TNC854.0 CD34^+^: 330.0	TNC: 20.0 × 10^7^; CD34^+^: 4.0 × 10^5^	TNC: 50.0 × 10^7^; CD34^+^: 175.0 × 10^5^	TNC: 70.0 × 10^7^; CD34^+^: 182 × 10^5^	17/17	Neutrophils: 15 d. Platelets: 49 d	Inhibition of differentiation by antagonization of AhR
UM171^37^	UM171, SCF, TPO, FL	7 d	TNCNA CD34^+^: 35.4	NA	TNC: 2.92 × 10^7^; CD34^+^: 28.8 × 10^5^	NA	22/22	Neutrophils: 18 d. Platelets: 42 d	Inhibition of erythroid and megakaryocytic differentiation
Hyperbaric oxygen^38^	NA	90 minutes	NA	NA	NA	NA	14/14	Neutrophils: 15 d. Platelets: 36.5 d	Reduction of host systemic erythropoietin

Abbreviations: AhR, aryl hydrocarbon receptor; C3a, completement 3a fragment; CXCR4, CXC chemokine receptor 4; dmPGE2, 16,16-dimethyl-prostaglandin E2; DPP4, dipeptidyl peptidase 4; FBS, fetal bovine serum; FL, FMS-like tyrosine kinase 3 ligand; G-CSF, granulocyte colony-stimulating factor; IBMT, intrabone marrow transplant; IL, interleukin; MGDF; megakaryocyte growth and development factor; MSC, mesenchymal stromal cell; NA, not applicable; SCF, stem cell factor; SDF-1, stromal derived factor-1; SIRT-1, silent mating type information regulation 2 homolog 1; SR-1, stem-regenin 1; TNC, total nucleated cell; TEPA, tetraethylenepentamine; TPO, thrombopoietin; UCB, umbilical cord blood.

### Expansion of UCB

#### Cytokine-based Expansion

Early work that aimed at enhancing UCB engraftment focused on the cytokines that promote HSPC survival and self-renewal.^39,40^ In these settings, UCB was cultured ex vivo in growth medium containing a combination of cytokines, often including stem cell factor (SCF), thrombopoietin (TPO), FMS-related tyrosine kinase 3 ligand (FL), interleukin-3 (IL-3), interleukin-6 (IL-6), granulocyte colony-stimulating factor (G-CSF) and granulocyte-macrophage colony-stimulating factor (GM-CSF), to allow for proliferation of HSCs.^41-44^ The challenge with this strategy is that it is difficult to control the concentration and time-window of individual cytokines in real time; as a result, differentiation was promoted, and transplantable HSCs did not increase in number. In a clinical trial^24^ in which UCB was expanded for 10 days with a combination of SCF, G-CSF and megakaryocyte growth and development factor (MGDF), 56.0- and 4.0-fold increases in total cell and CD34^+^ cell numbers, respectively, were achieved. However, there was no improvement in neutrophil and platelet recovery. Besides, higher rates of acute (66.7%) and chronic (74.0%) GVHD were observed in the patients than those of contemporary studies,^24^ reflecting a change in HSPC biology after expansion.

#### Copper Chelator-mediated Expansion

One clinical trial was based on the observation that supplementation with tetraethylenepentamine (TEPA), a copper chelator, reduced differentiation and increased engraftment of UCB CD34^+^ cells in NOD/SCID mice.^45^ In this trial,^25^ UCB was split into 2 portions, one in which CD133^+^ cells were selected and subsequently expanded with growth medium containing TEPA, SCF, FL, IL-6, TPO for 21 days; the other portion was not manipulated. With this regimen, patients received an unmanipulated portion and subsequently expanded portions. The fold expansion of CD34^+^ cells was 6.0, and the median time to neutrophil and platelet recovery was 30 and 48 days, respectively. Since this trial, 2 other phase II/III trials (NCT00469729 and NCT01484470) have been completed in multiple centers. For 101 patients who received TEPA-expanded UCB units, faster median neutrophil (21 days vs 28 days) and platelet (54 days vs 105 days) recovery was achieved compared with 295 patients receiving unmanipulated double UCB units (NCT00469729).^26^ There was also an advantage in 100-day survival for TEPA group compared with unmanipulated group (84.2% vs 74.6%). In this case, acute and chronic GVHD rates were similar between both groups. Notably, in this trial, an amazing 77.0-fold increase in CD34^+^ number was reported for a 21-day culture, but the cause for the difference from the previous phase I trial (6.0-fold increase) was not clear.

#### Notch-mediated Expansion

Activation of Notch signaling via surface Notch receptors promotes HSC self-renewal.^46,47^ In a preclinical study,^46^ it was found that among various Notch ligands, an immobilized form of DLL1 (DLL1^ext-IgG^) performed best at UCB HSC expansion. A clinical trial^28^ in which UCB units were cultivated with DLL1^ext-IgG^ for 16 days reported a 164.0-fold increase in CD34^+^ cell number. After receiving one unmanipulated and one DLL1^ext-IgG^-expanded UCB unit, patients showed a markedly shortened median time to engraftment (16 days). Rapid myeloid recovery in recipients of DLL1^ext-IgG^-expanded UCB units also occurred in the early period, at 7 days post-UCBT. Nevertheless, only one of 10 patients exhibited an expanded cell population in the engraftment at 1 year posttransplantation, indicating a lack of long-term HSCs after DLL1^ext-IgG^-mediated expansion.^47^ In addition, all patients experienced grade II GVHD, a significantly higher rates than those of conventional studies.

#### Nicotinamide-mediated Expansion

Nicotinamide is a precursor to nicotinamide adenine dinucleotide (NAD) and inhibits SIRT1 activity,^48^ and addition of nicotinamide prevents UCB CD34^+^ cells from differentiating.^49^ A clinical product, NiCord, has been developed and applied in phase I and II trials. A pilot study^33^ with 11 participants found that the median time to neutrophil and platelet recovery was shortened to 13 and 33 days, respectively, when using NiCord and unmanipulated UCB units. Only 5 patients experienced grade II GVHD in this trial. Moreover, one patient had mixed chimerism at 3 years posttransplantation, suggesting the existence of long-term HSCs in the Nicord portion. Based on this observation, another trial^34^ evaluated the effect of a single NiCord infusion on UCBT. Among 36 participants, 34 showed engraftment, and the median time to neutrophil and platelet recovery was markedly shortened (11.5 and 34 days, respectively). Importantly, lower rates of acute (44% versus 56%) GVHD were observed in patients with NiCord infusion than those in comparator cohort.

#### SR-1-medieated Expansion

SR-1 is a purine derivative identified by a high-throughput screen to promote human HSC expansion via inhibition of aryl hydrogen receptor (AhR) signaling.^50^ A clinical trial^36^ based on a double UCB platform with one SR-1-expanded and one unmanipulated UCB unit was recently completed (NCT01474681). In this trial, UCB CD34^+^ cells were isolated and cultured with SR-1, SCF, TPO, and FL for 15 days and then infused into recipients together with CD34^−^ potion and an unmanipulated UCB unit. This strategy resulted in HSC expansion on a large scale, with an 854.0-fold increase in total nucleated cells and 330.0 in CD34^+^ cells. In all 17 patients, engraftment occurred, with a significant improvement in the median time to neutrophil (15 days) and platelet (49 days) recovery. In terms of GVHD, the rates of acute and chronic GVHD in SR-1 group were similar to historical cohorts.

#### UM171-mediated Expansion

UM171 is a pyrimidoindole derivative that promotes UCB long-term HSC expansion independent of AhR signaling.^51^ The presence of UM171 leads to inhibition of erythroid and megakaryocyte expression.^51^ Mechanistic studies demonstrate that UM171 effectively targets RCOR1, LSD1, and HDAC2 through CRL3/KBTBD4 complex by proteasomal degradation, which further re-establishes H3K4me2 and H3K27ac epigenetic marks to prevent HSC attrition during in vitro culture.^52^ It has also been reported that UM171 treatment selectively increases EPCR^+^ or ITGA3^+^ cell populations, which possess higher long-term repopulation ability than the EPCR^-^ or ITGA3^−^ component.^53,54,55^ In a recently completed trial,^37^ 22 patients who received a single 7-day UM171-expanded UCB unit experienced a marked shortening of the median time to neutrophil (18 days) and platelet (42 days) recovery. Notably, patients infused with UM171-expanded UCB also had less GVHD occurrence than those infused with double unmanipulated UCB units. In light of this observation, many more UM171-related trials have begun in multiple centers.

#### Mesenchymal Stromal Cell (MSC)-mediated Expansion

To mimic the BM niche that supports HSC self-renewal, researchers have cultured UCB CD34^+^ cells on STRO-3^+^ MSCs supplemented with SCF, TPO, FL, and G-CSF.^29^ In this clinical trial (NCT00498316), total nucleated cell and CD34^+^ cell numbers were increased 12.2- and 30.1-fold, respectively, at 14 days postculture. Participants received one cocultured and one unmanipulated UCB unit successively, and 23 of the 24 recipients showed mixed chimeras, with a markedly shortened median time to recovery of neutrophils (15 days) and platelets (42 days). The rate of acute GVHD was 55%, comparable to that of historical cohorts. However, unmanipulated UCB units prevailed in all patients’ chimera at 1-year posttransplantation, suggesting that the engrafting ability was impaired in cocultured UCB units.

### UCB Homing Promotion

#### Prostaglandin-mediated Homing

A long-activating derivative of prostaglandin E2, 16,16-dimethyl-PGE2 (dmPGE2), has been identified as increasing the long-term HSC number in zebrafish, mice, and humans via enhancement of Wnt signaling and promotion of survival; dmPGE2 also upregulates expression of CXCR4, which is a homing receptor of HSCs to the BM niche.^56-58^ In a pilot study,^30^ 12 participants who received one dmPGE2-pulsed UCB unit and one unmanipulated UCB unit displayed promising improvement in hematopoietic engraftment, and the median time to neutrophil (17.5 days) and platelet (43 days) recovery was shortened. Interestingly, 10 of the 12 patients showed 100% engraftment with dmPGE2-pulsed UCB units, suggesting a functional boost of HSC engraftment ability with dmPGE2 treatment. In terms of GVHD, only 4 patients experienced mild GVHD symptom.

#### C3a-mediated Homing

C3a is a component of the completement system. C3a can be produced by BM stromal cells.^59^ It was found that C3a is able to promote CD34^+^ cell migration toward SDF-1 via the surface C3a receptor in vitro, acting through the SDF-1-CXCR4 axis to facilitate HSC homing to BM.^59,60^ In one clinical trial,^31^ the UCB unit was primed with C3a for 15 minutes and then infused into participants along with one unmanipulated UCB unit. Strikingly, the median time to neutrophil recovery with C3a priming was 7 days. However, only 9 of the 29 patients showed engraftment skewing toward C3a-primed units, suggesting that C3a priming may act on short-term repopulating cells rather than long-term HSCs.

#### DPP4-mediated Homing

Dipeptidyl peptidase-4 (DPP4) cleaves SDF-1 at the N-terminus to generate a truncated form that competitively blocks the binding of wild-type SDF-1 to CXCR4, thus impairing HSC homing.^61^ Sitagliptin is an oral drug that inhibits DPP4 activity and enhances HSC engraftment in mouse models.^62^ In a clinical trial,^32^ participants received 600 mg sitagliptin once a day before UCBT until 2 days afterward. However, the outcome was not exciting, with only modest improvement in the median time to neutrophil recovery (21 days). Anyway, the incidence of GVHD was rare in this trial, with only one patient experiencing grade II GVHD.

#### Fucosylation-mediated Homing

Preclinical studies have shown that fucosylation of UCB cells can augment P-selectin and E-selectin binding of HSCs and thereby enhance homing to BM.^63,64^ In a clinical trial,^35^ UCB units were incubated with fucosyl-transferase VI in vitro for 30 minutes to allow fucosylation and then infused into patients together with unmanipulated UCB units. Twenty of 22 recipients showed engraftment, with a markedly shortened median time to neutrophil (27 days) and platelet (26 days) recovery. Besides, there was not increase in GVHD incidence in this trial.

#### Intrabone Marrow Transplantation

It has been reported that only 10-15% of HSCs successfully home to the BM niche after intravenous infusion.^65^ Intra-BM transplantation in a mouse model showed 10-fold efficiency of intravenous transplantation in marrow repopulation.^66^ Based on these observations, a clinical trial was conducted to transplant a UCB unit intra-BM.^27^ All 28 patients exhibited mixed chimeras after UCBT, but modest improvement in the median time to neutrophil (23 days) and platelet (36 days) recovery occurred.

#### Hyperbaric Oxygen Therapy (HBT)

UCB CD34^+^ cells display an impaired ability to migrate to the BM niche after exposure to erythropoietin (EPO),^67^ but lowering the EPO concentration by HBT can facilitate UCB HSC homing to the BM niche in a mouse model.^67^ In a clinical trial,^38^ patients received HBT before single- unit UCBT, and the median time to neutrophil (15.0 days) and platelet (36.5 days) recovery was significantly improved. However, overall survival did not differ from that of conventionally treated patients.

### Recent Progress in Preclinical Studies on UCB Engraftment

In a normal physiological environment, self-renewal of HSPCs is regulated by complex regulatory networks of intrinsic factors (such as transcription factors, cell cycle regulators, and various metabolic pathways) as well as external cues.^68^ Supportive cells in the BM niche can produce a variety of cell growth factors, such as SCF, TPO, TGF-β1, and G-CSF, and provide cell-cell contact signals to affect HSC behavior.^15,16^ In general, these extrinsic signals are transmitted by surface or intracellular receptors and adaptors and converge to a regulatory core comprising key transcription factors (Fig. 1). The delicate balance between self-renewal and differentiation is determined by these core regulators. Numerous studies have sought to promote HSC self-renewal versus differentiation via certain stimuli. Recent progress in preclinical studies may help to improve UCB engraftment in the clinical setting (summarized in Table 3).

**Table 3.** Recent progress in preclinical studies of UCB engraftment improvement

Agents	Input cells	Culture media	Culture period	Result	Possible mechanism	Reference
Small molecules						
5azaD/TSA	CD34^+^	Serum-containing media, SCF, TPO, FL, IL-3	9 d	7.0-fold increase in SRCs	Hypomethylation of HSC self-renewal genes	^69^
VPA	CD34^+^	Serum-containing media, SCF, TPO, FL, IL-3	9 d	Maintenance of SRCs	Hypomethylation of HSC self-renewal genes	^69^
VPA	CD34^+^	Serum-free media, SCF, TPO, FL, IL-3	7 d	36.0-fold increase in SRCs	Upregulation of pluripotency genes and CXCR4, increased aldehyde dehydrogenase activity	^70^
LMK235	CD34^+^	RPMI, 10% FBS, SCF, TPO, FL	16 h	6.0-fold increase in SRCs	Inhibition of HDAC5, increased CXCR4 surface expression	^71^
Garcinol	CD34^+^	StemSpan SFEM, SCF, TPO, FL	7 d	2.5-fold increase in SRCs	Inhibition of HATs	^72^
CPI203	CD133^+^	StemSpan SFEM, SCF, TPO, FL	5 d	Improved BM engraftment at 21 weeks post-transplantation	Inhibition of BET, promotion of megakaryocyte differentiation	^73^
SB203580	CD133^+^	StemSpan H3000, SCF, TPO, FL	7 d	Improved BM engraftment at 10 weeks post-transplantation	Inhibition of the p38/MAPK pathway, upregulation of CXCR4	^74^
C7	MNC	StemSpan SFEM, SCF, TPO, FL, IGFBP-2	11 d	2.5-fold increase in SRCs	Inhibition of the p38/MAPK pathway	^75^
JNK-IN-8	CD34^+^	StemSpan SFEM, SCF, TPO, FL,	10 d	3.9-fold increase in SRCs	Inhibition of the JNK pathway	^76^
JNK-IN-8	CD34^+^	StemSpan SFEM, SCF, TPO, FL,	24 h	13.5-fold increase in SRCs	Inhibition of the JNK pathway	^77^
Baclofen	CD34^+^	NA	2 h	Improved BM engraftment at 16 weeks post-transplantation	Agonization of GABA signaling, promotion of B-cell differentiation	^78^
Resveratrol	CD34^+^	StemSpan SFEM, SCF, TPO, FL, IL-6	9 d	Improved engraftment of primary and secondary transplants	Reduction of oxidative stress	^79^
4HPR	Lin^−^MNC	StemPro, SCF, TPO, FL, IL-3, IL-6, GM-CSF	8 d	2.5-fold increase in SRCs	Inhibition of DEGS1, activation of ER stress and autophagy, reduction of ROS	^80^
GW9662	CD34^+^	RPMI, 10% FBS, SCF, TPO, FL	4 d	5.0-fold increase in SRCs	Antagonization of PPAR-γ signaling, enhancement of glycolysis	^81^
Angiogenin	CD34^+^	StemSpan SFEM	2 h	8.9-fold increase in SRCs	Promotion of tiRNA production and quiescence of HSPCs	^82^
NOV/CCN3	CD34^+^	StemSpan SFEM, SCF, TPO, FL,	8 h	6-fold increase in SRCs	Recruitment of otherwise nonfunctioning HSCs	^83^
OAC1	CD34^+^	RPMI, 10% FBS, SCF, TPO, FL	4 d	3.5-fold increase in SRCs	Induction of OCT4 expression	^84^
Fed-batch system	Lin^−^MNC	IMDM, 20% BIT serum substitute, SCF, TPO, FL	12 d	11.0-fold increase in SRCs	Global maintenance of subthreshold levels of inhibitory factors	^85^
Cellular niches						
Revitalized MSCs (transduced with Klf7, Ostf1,Xbp1, Irf3 and Irf7)	CD34^+^	StemSpan SFEM, 10% KSR, TPO	6 d	5.7-fold increase in SRCs	Reduced DNA damage	^86^
Extracellular matrices						
Zwitterionic hydrogel	CD34^+^	StemSpan SFEM II, SCF, TPO, FL, IL-6, IL-3	24 d	78.0-fold increase in SRCs	Reduced metabolic activity, avoidance of ROS production, p38α, mTORC and p16INK activation	^87^
3D fibrin/collagen/PCL scaffolds with/without MSC	CD34^+^	StemSpan, SCF, TPO, FGF-1, ANGPTL-5, IGFBP-2	7 d	Improved BM engraftment at 12 weeks post-transplantation	Unclear	^88^
PES nanofiber	CD34^+^	StemSpan, SCF, TPO, FL, IL-3	10 d	Improved BM engraftment at 6 weeks post-transplantation	Unclear	^89^
Combination						
LY2228820, rapamycin, SR-1	CD34^+^	StemSpan SFEM, SCF, TPO, FL, IL-6	7 d	4.1-fold increase in SRCs	Coinhibition of p38α, mTORC1 and AhR signaling, reduction of senescence	^90^
4HPR, UM171, SR-1	Lin^−^MNC	StemPro, SCF, TPO, FL, IL-3, IL-6, GM-CSF	8 d	4.8-fold increase in SRCs	Inhibition of DEGS1, AhR signaling and erythroid and megakaryocyte differentiation	^80^
MLLT3 overexpression, UM171, SR-1	CD34^+^	StemSpan SFEM II, SCF, TPO, FL	15 d	12.5-fold increase in SRCs	Protection of HSC stemness program through DOT1L and H3K79me2	^91^

Abbreviations: 4HPR, *N*-(4-hydroxyphenyl) retinamide; 5azaD/TSA, 5aza-2ʹ-deoxycytidine/trischostatin A; AhR, aryl hydrocarbon receptor; BET, bromodomain and extraterminal domain; CXCR4, CXC chemokine receptor 4; DEGS1, Delta 4-desaturase, sphingolipid 1; DOT1L, DOT1-like histone H3K79 methyltransferase; FBS, fetal bovine serum; FGF-1, fibroblast growing factor-1; FL, FMS-like tyrosine kinase 3 ligand; GABA, γ-aminobutyric acid; GM-CSF, granulocyte-macrophage colony-stimulating transcription factor 4; HAT, histone acetyltransferase; HDAC, histone deacetylase; IL, interleukin; JNK, c-Jun N-terminal kinase; MAPK, mitogen-activated protein kinase; MLLT3, myeloid/lymphoid or mixed-lineage leukemia (trithorax homolog, Drosophila) translocated to, 3; MNC, mononuclear cell; MSC, mesenchymal stromal cell; mTORC1, mammalian target of rapamycin complex 1; OCT4, octamer-binding transcription factor 4; PES, polyethersulfone; PPAR, peroxisome proliferator-activated receptor; ROS, reactive oxygen species; SCF, stem cell factor; SRC, SCID-repopulating cells; SR-1, stem-regenin 1; TPO, thrombopoietin; VPA, valproic acid.

#### Epigenetic Modifiers

De novo transcription of genes is regulated by local chromatin accessibility through epigenetic modification, which has been demonstrated to play a key role in cell fate determination in various cell types.^92,93^ A recent study reported that after 9 days of culture, the DNA methyltransferase inhibitors 5azaD and TSA increased the number of SCID-repopulating cells (SRC) among UCB CD34^+^ cells by 7.0-fold.^69^ Additionally, the mechanistic study found a marked decrease in methylation levels at HSC self-renewal gene loci after 5azaD and TSA treatment. Surprisingly, another agent, the HDAC3 inhibitor VPA, led to SRC number maintenance but not increase, which seems contradictory to other reports.^69,70^ This result may have been caused by certain substances in serum that blocked the function of VPA, as serum-free culture of UCB CD34^+^ cells with VPA increased the SRC number by 36.0-fold after 7 days.^70^ Moreover, VPA was found to upregulate expression of the homing receptor CXCR4 and activity of ALDH in HSCs. In addition to HDAC3 inhibition, HDAC5 inhibition has been shown to improve UCB engraftment with the SRC number increasing 6.0-fold after 16 hours of treatment with the HDAC5 inhibitor LMK235, an effect that might be mediated by enhanced NF-kB activity and upregulated CXCR4 expression.^71^ Interestingly, a recent report^52^ demonstrated that UM171 was involved in epigenetic regulation of HSCs by targeting the components of CoREST complex (RCOR1, LSD1, HDAC2) for degradation. In this study, the HDAC inhibitor panobinostat selectively expanded CD34^+^EPCR^+^ HSCs after 4-day culture. Despite lacking of function assay, this study highlights the role of epigenetic regulation to orchestrate HSC activity. Other epigenetic modulators, such as histone acetyltransferase (HAT) and the bromodomain and extraterminal domain (BET) family, have also been reported to participate in HSC self-renewal. Garcinol, an inhibitor of HATs, caused a 2.5-fold increase in SRC number,^72^ and CPI-203, a BET inhibitor, enhanced UCB HSC engraftment by promoting megakaryocyte differentiation, possibly with an effect on megakaryocyte fate-primed HSCs which are at the top of the hematopoietic hierarchy.^73^ Overall, epigenetic modifiers play vital roles in HSC engrafting ability by selectively adjusting transcription factor activity on the self-renewal or differentiation network.

#### MAPK Superfamily

The mitogen-activated protein kinase (MAPK) superfamily is heavily involved in proliferation and various physiological processes, classified into the p38 MAPK, c-Jun N-terminal kinase (JNK) and extracellular signal-regulated kinase (ERK) signaling pathways.^94^ SB203580, a p38 inhibitor, led to modest enhancement in the case of UCB CD133^+^ cell engraftment.^74^ A more recent study screened out a structure-modified molecule named C7, causing a 2.0-fold increase in SRC number in UCB mononuclear cells.^75^ Furthermore, JNK has recently been proven to participate in HSC stemness and self-renewal regulation.^76,77^The inhibition of JNK activity by JNK-IN-8 increases the SRC number among UCB CD34^+^ cells by 3.9 folds at 10 days post-culture.^76^ Moreover, a greater increase in SRC number was achieved by shortening the culture period to 24 hours and increasing the JNK-IN-8 concentration.^77^ Interestingly, the presence of a JNK inhibitor during HSC collection was also found to enhance long-term HSC recovery from UCB units.^77^ Mechanistically, JNK inhibition promotes self-renewal gene expression and prevents metabolic activation in HSCs.^77^

#### Metabolic Regulation

It has been observed frequently that long-term HSCs are more metabolically inactive than are short-term HSCs.^95-97^ Low-level metabolic activity can reduce metabolite-related reactive oxygen species (ROS) accumulation and endoplasmic reticulum (ER) stress, thus maintaining HSC survival and stemness.^98,99^ In recent years, there have been various attempts to target metabolic regulation, including glycolysis and lipid metabolism. Resveratrol is a nonflavonoid polyphenol that activates SIRT1, which plays an essential antioxidative role.^100^ Activated SIRT1 in turn upregulates FOXO3a and then degrades cellular ROS.^100^ A recent study^79^ found that addition of resveratrol enhances serial engraftment of UCB CD34^+^ cells after 9 days of culture, with a comparable effect to SR-1. However, the specific number of SRCs was not calculated. Another study^80^ reported an important role for lipid metabolism in HSC self-renewal regulation. DEGS1 is a sphingolipid enzyme with low expression in long-term HSCs but high expression in differentiating progenitors.^80^ It was observed that DEGS1 expression in UCB CD34^+^ cells was rapidly upregulated upon culture in vitro, with the SRC number increasing by 2.5-fold in the presence of the DEGS1 inhibitor N-(4-hydroxyphenyl) retinamide (4HPR).^80^ Such enhancement of HSC engraftment may result from ER stress alleviation, autophagy promotion and ROS reduction. Notably, a greater increase in SRC number can be achieved by combining 4HPR with UM171 and SR-1.^80^ In the case of glycolysis modulation, the PPARγ inhibitor GW9662 achieved a 5.0-fold increase in SRC number by enhancing glycolysis and suppressing lipid metabolism.^81^ These observations suggest that promoting quiescence is beneficial for HSC stemness preservation. Other agents, for instance, angiogenin, promote HSC quiescence by enhancing tiRNA production in HSCs,^82^ and NOV/CCN3 is reported to increase the HSC number via cell cycle inhibition and metabolic suppression.^83^

#### Extracellular Matrix

The ECM is an important component that provides signals to regulate HSC behavior. Indeed, the ECM has a significant effect on biological processes such as cell differentiation, proliferation, adhesion, morphogenesis, and phenotypic expression.^39^

BM-derived MSCs have been demonstrated to provide certain signals for HSCs to proliferate and self-renew in both clinical trials^29^ and preclinical studies.^86,101^ Nevertheless, MSCs are not easily produced on large scales, and there are difficulties in quality control. Therefore, a more defined culture method is needed to facilitate translation to the clinic. Many biomaterials have been tested for their ability to enhance HSC self-renewal, but most of them have failed.^13^ Even the most efficient methods, such as 3D fibrin/collagen/PCL scaffolds^88^ and PES nanofibers,^89^ led to only a modest increase in short-term engraftment. However, strikingly, a recent report^87^ indicated that zwitterionic hydrogel markedly increased the SRC number among UCB CD34^+^ cells by 78.0-fold after a 24-day 3D culture via multiple signaling pathways, including reduced metabolic activity and avoiding ROS production and p38α, mTORC and p16INK activation. This study suggested that fine-tuned orchestration of diverse cues is important to achieve better regulation of the HSC core network.

## Discussion

Clinical trials have demonstrated the promising translational potential of ex vivo manipulation of UCB to improve HSC engraftment. Regardless, more endeavors are needed to explore more efficient strategies, as many methods still rely on double UCB platforms, except for NiCord and UM171, the clinical benefit of which requires further evaluation. Despite many positive results using preclinical mouse models, there is low translational efficiency to human counterparts of proposed protocols. In general, regulation of human HSCs differs in some aspects from mouse HSCs,^50,102^ and even HSCs from different sources show different transcriptional profiles.^103,104^ Additionally, it is challenging to sophistically control the agent concentration and time-window of ex vivo manipulation.^85^ In fact, the regulatory core of HSC self-renewal remains unclear. RNA-seq and proteomic analysis may help identify novel regulators.^80,91,105^ At present, the combination of different methods might help to maximize the HSC engraftment potential by orchestrating multiple cues.^80,90,91^ Overall, much work is needed to optimize UCB engraftment.

## Data Availability

The data that support the findings of this study are available from the corresponding author upon reasonable request.
